# S100A8 alarmin supports IL-6 and metalloproteinase-9 production by fibroblasts in the synovial microenvironment of peripheral spondyloarthritis

**DOI:** 10.3389/fimmu.2022.1077914

**Published:** 2023-01-09

**Authors:** José L. Arias, Samanta C. Funes, Rodrigo Blas, Eduardo Callegari, Ricardo J. Eliçabe, María D. Páez, Alicia Munarriz, Rodolfo Pardo-Hidalgo, Héctor Tamashiro, María S. Di Genaro

**Affiliations:** ^1^ Biochemistry Department, Universidad Nacional de San Luis, San Luis, ;Argentina; ^2^ Instituto Multidisciplinario de Investigaciones Biológicas-San Luis (IMIBIO-SL), Consejo Nacional de Investigaciones Científicas y Técnicas (CONICET)-Universidad Nacional de San Luis (UNSL), San Luis, Argentina; ^3^ Centro Médico MEDICI, San Luis, Argentina; ^4^ South Dakota (SD) Biomedical Research Infrastructure Network (SD BRIN), Stanford School of Medicine, University of South Dakota, Vermillion, SD, United States; ^5^ Centro Médico Centro de Especialidades Neurológicas y Rehabilitación (CENYR), San Luis, Argentina; ^6^ Centro de Rehabilitación Médica Centro de Rehabilitación Médica (CER), San Juan, Argentina; ^7^ Clínica Bolívar, San Luis, Argentina

**Keywords:** S100A8, synovial fluid, fibroblast-like synoviocytes, IL-6, spondyloarthritis, MMP-9

## Abstract

**Introduction:**

Spondyloarthritis (SpA) is a common autoinflammatory disease. S100A8/ S100A9 alarmin is strongly expressed in the synovial sublining layers of psoriatic arthritis. S100A8/ S100A9 is the most abundant protein in rheumatoid arthritis synovial fluid (SF) and has a key role in promoting IL-6 expression in fibroblast-like synoviocytes (FLS). The molecular mechanisms and the role of S100-alarmins in the synovial microenvironment of SpA have never been demonstrated.

**Methods and Results:**

Here, we confirm the effect of the synovial microenvironment of peripheral SpA on interleukin-6 (IL-6) and metalloproteinase (MMP)-9 production by FLS. MMP-9 expression and activity were detected, which were reduced in the presence of anti-IL-6R. Analyzing cell signaling mechanisms, we found that stimulation with IL-6 co-triggered MMP-9 and IL-10 secretion. MMP-9 secretion depended on JNK and p38 MAPKs, whereas IL-10 secretion was dependent on the JAK pathway as a potential feedback mechanism controlling IL-6-induced MMP-9 expression. Using a proteomic approach, we identified S100A8 in the peripheral SpA SF. This presence was confirmed by immunoblotting. S100A8 increased the IL-6 secretion via ERK and p38 MAPK pathways. Furthermore, anti-S100A8/A9 reduced both IL-6 and MMP-9 production induced by SpA SF in FLS.

**Discussion:**

Our data reveal a marked relationship between S100A8 alarmin with IL-6 and MMP-9 secretion by FLS in the real synovial microenvironment of peripheral SpA. These results identify a mechanism linking S100A8 to the pathogenesis of peripheral SpA.

## Graphical Abstract

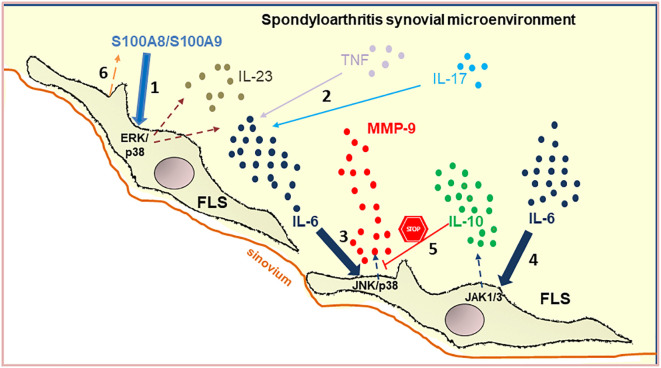

## 1 Introduction

Spondyloarthritis (SpA) is a group of inflammatory diseases classified into axial and peripheral SpA (pSpA) (1, 2). The pSpA classically comprises psoriatic arthritis (PsA), reactive arthritis (ReA), SpA related to inflammatory bowel disease (IBD-SpA), and undifferentiated SpA (uSpA) (1). Like other chronic arthropathies, such as rheumatoid arthritis (RA) and osteoarthritis (OA), SpA constitutes a public health problem (3). However, the biological factors and molecular mechanisms underlying pSpA progression remain unclear.

In healthy joints, the synovium is a delicate structure where fibroblast-like synoviocytes (FLS) provide nutritive proteins, lubricating molecules and control physiologic extracellular-matrix remodeling by secretion of matrix-degrading enzymes (MMPs) and several matrix components (4). Furthermore, dysregulated FLS leads to the onset and progression of arthritis, including SpA (5). In addition, synovial fluid (SF) is a plasma filtrate located in the joints that receives protein from surrounding tissues: articular cartilage, synovial membrane and bone (6). Therefore, the composition of the SF is very complex and strongly influences the joint microenvironment (7). Synovial fluid augments the FLS response to ligands of Toll-like receptors (TLRs) (8), which enhance their pathogenic behavior (9). Hence, the SF represents an inseparable element of arthritis (7, 10).

Interleukin-(IL)-6 is a pleiotropic cytokine expressed by a variety of immune and non-immune cells, such as FLS (11). IL-6 is one of the most studied biomarkers in SpA (12). The biological responses of IL-6 are mediated by the membrane-bound IL-6R (IL-6Rα), which is expressed in a few cell types. The IL-6/IL-6Rα complex engages trans-membrane gp130 (IL-6Rβ; CD130), triggering the binding and phosphorylation of JAK, which then phosphorylates gp130 and leads to the activation of STAT and mitogen-activated protein kinases (MAPKs) signaling pathways (13). Virtually all cell types express gp130, which can bind soluble IL-6/IL-6Rα complexes (i.e. trans-signaling). Moreover, the SF of patients with arthritis contains high levels of IL-6 and sIL-6R (14), sustaining trans-signaling. In addition, the induction of MMP-9 expression in macrophages is a direct mechanism by which IL-6 can contribute to the pathogenesis of chronic arthritis (13). MMP-9 is a gelatinase able to cleave denatured collagen (gelatin), type IV collagen, and native type I and II collagens which mainly exist in cartilage (15). MMP-9 is released as pro-enzyme (pro-MMP-9), and it is activated to mature MMP-9 expressed in three major forms: monomer, homodimer and a complex with neutrophil gelatin-associated lipocalin (NGAL) (16). MMP-9 is low or absent in most normal tissues (17). However, MMP-9 increases in synovial fluid of SpA patients (10), MMP-9 activity correlates with SpA severity (18), and it participates in the pathogenesis of arthritis (19).

On the other hand, alarmins or danger-associated molecular patterns (DAMPs) are endogenous molecules rapidly released into the extracellular milieu by stressed, injured or necrotic cells and activated immune cells (20). S100 proteins, such as S100A8 and S100A9, are among the best-known alarmins (20, 21). S100A8 and S100A9 form a non-covalent heterodimer S100A8/S100A9, which increases extracellularly in inflammatory disease including PsA (21), and induces the secretion of multiple cytokines that sustain and exacerbate inflammation (21, 22).

Despite intensive research, pathological mechanisms that explain the change of FLS to an altered phenotype in peripheral SpA remain largely unknown. We hypothesized that in the synovial microenvironment, FLS encounter pathological signals that promote the secretion of inflammatory mediators by these cells. To test this hypothesis, we investigated the role of the synovial microenvironment in IL-6 and MMP-9 production by FLS, and then, the effect of S100A8, an alarmin that we found in the SpA synovial fluid through proteomic analysis. The molecular mechanisms were also explored. Although extracellular S100A8 of SpA synovial microenvironment is not the unique stimulus for the inflammatory response of FLS, this alarmin induced significant IL-6 production by FLS, which is associated with MMP-9 secretion, indicating that alarmins are playing a role during peripheral SpA inflammation.

## 2 Methods

### 2.1 Patients’ synovial fluid

SFs were obtained from patients with psoriatic arthritis (PsA, 6), reactive arthritis (ReA, 2), and undifferentiated SpA (uSpA, 1). The patients were classified according to the Assessment of SpondyloArthritis International Society criteria for pSpA (2). The characteristics of the patients were: medium age, 48 ± 19 years; gender (male:female), 6:3; and disease duration, 1 month-3 years. The SF of pSpA were pooled (n=9) for analysis as this method has been shown to reduce inter-individual differences (23). In addition, pools of SF of RA [n=10; medium age, 54 ± 13; gender (male:female), 1:9] and OA [n=9; medium age, 73 ± 10; gender (male:female), 3:6] patients were used for comparative purpose. All RA patients fulfilled the American Rheumatism Association criteria for RA (24). All the patients who participated in the study signed a written consent. The protocol followed in this study (CE002-2017) was approved by the Ethics Committee of the Institute of Biology and Experimental Medicine (IBYME, CONICET, Buenos Aires, Argentina).

### 2.2 Cell culture

Primary FLS were isolated from the SF of 3 patients as previously described (25) and cultured in Dulbecco’s modified Eagle medium (DMEM, Thermo Fisher Scientific, San Diego, CA, US) supplemented with 10% fetal bovine serum (FBS), 2 mM L-glutamine, 1 mM pyruvate, 100 IU/ml penicillin and 100 μg/ml streptomycin (Thermo Fisher Scientific). The SW982 cell line of human FLS was gently provided by Dr. Victoria Delpino (National University of Buenos Aires, Argentina). The cells were cultured at 37°C in a humidified atmosphere with 95% air and 5% CO2. The FLS cultures were found to be completely free of other cells as assessed by morphology (Giemsa staining) and specific immunofluorescence staining with an anti-prolyl-4-hydroxylase antibody (prolyl-4-hydroxylase mAb, Acris GMbH, Hiddenhausen, Germany) (25). The FLS from passages 4-6 were used for subsequent experiments.

### 2.3 Stimulation of FLS

FLS were seeded into 24-well plates (2x10^4^ cells/well) and stimulated with pSpA, RA or OA SF pool at dilution 1:10 in DMEM medium without fetal bovine serum for 72 h. In some experiments, the FLS were stimulated for 48h with pSpA pool in the absence or the presence of anti-IL-6 receptor (tocilizumab, 200 μg/ml), anti-TNF (infliximab, 200 μg/ml), TNF inhibitor (etanercept, 100 μg/ml), anti-IL-17 (sekukinumab, 100 μg/ml) or anti-human S100A8/S100A9 mouse IgG (Ultra-LEAF, Biolegend, San Diego, CA, USA, 5 μg/ml). To study the signaling pathways, FLS were pretreated with JNK (SP600125), ERK (PD98059), or p38 MAPK (SB203580) inhibitors (all from Calbiochem, San Diego, CA, USA,10 μM), or JAK1/3 inhibitor (tofacitinib, 1000 nM), or 0.1% DMSO (diluent control) for 1 h and additionally stimulated with pSpA pool, as previously described. Different concentrations of IL-6 (5-500 pg/ml, BD Biosciences, San Diego, CA, USA) or S100A8 (1-10 ng/ml, Biolegend, San Diego, CA, USA) were also assayed. In addition, experiments using FLS stimulated with SF treated with hyaluronidase (50 μg/ml, Sigma-Aldrich; Merck KGaA, Darmstadt, Germany) (SpA-H), or proteinase K (500 μg/ml, Promega, Madison, WI, USA) (SpA-P) were performed.

### 2.4 Cytokine and MMP-9 quantification

IL-6, IL-10 and MMP-9 levels were determined in cell culture supernatants using Enzyme-linked immunosorbent assay (ELISA) kits, according to the manufacturer’s instructions. The IL-6 kit was obtained from BD Biosciences, the IL-10 kit from eBioscience (San Diego, CA, USA), and the MMP-9 kit from Biolegend.

### 2.5 Gelatin-zymography

Culture supernatants were mixed with a non-reducing SDS gel sample buffer and applied without boiling to a 10% polyacrylamide gel containing 0.1% SDS and 2 mg/ml gelatin. After electrophoresis, the gels were washed in 50 mmol/l Tris-HCl (pH 7.5) containing 0.15 mol/l NaCl, 10 mmol/l CaC12, 0.02% NaN3, 2.5% Triton X-100 at room temperature, and then incubated in the same buffer with 1% Triton X-100 for 42 h (26). Proteins were stained with Coomassie Brilliant Blue R-250 and destained with 40% methanol and 10% acetic acid. The destained band area was measured in pixels for band intensity with the software ImageJ 1.45s (National Institutes of Health, Bethesda, MD, USA).

### 2.6 Proteomic study of peripheral SpA SF pool

Protein abundance in individual gels correlates with the pools derived from these individuals (27). Thus, a pool of SpA-H was analyzed. We used two different approaches to decrease the complexity of the SF dynamic range: 1) combined “Gel-Free” (GF) (in solution digestion), and 2) “Gel-LC” (GLC) (in gel digestion) upstream of the 2D-liquid chromatography-tandem mass spectrometry-based bottom-up analysis. For GF protocol, the sample was subjected to reduction and alkylation, followed by digestion with in-solution trypsin sequencing grade (Promega). The tryptic digested peptides were resuspended in 0.1% formic acid in water, injected into 2D-nanoAcquity UPLC (Waters, Milford, MA, USA), analyzed by tandem mass spectrometry using a Synapt G1 Q-Tof HDMS (Waters) and corroborated by Ultimate 3000RS nano UHPLC coupled to Q Exactive Plus Orbitrap MS (Thermo Scientific, Waltham, MA, USA), as previously described (28). For GLC, SDS-PAGE gels (10%) were stained using colloidal-Coomassie. Protein bands were excised and chopped. The gel pieces were destained with 50 mM of ammonium bicarbonate/acetonitrile (50/50% v/v), reduced with DTT, followed by alkylation with iodoacetamide. Then, in-gel trypsin digestion was carried out as previously described (28). The bioinformatics analysis was performed using Mascot Distiller v 2.6.2 and Mascot server 2.5.1 (www.matrixscience.com) in MS/MS ion search mode against SwissProt v2020_06 (563,972 sequences; 203,185.243 residues), with taxonomy filter for Homo sapiens (human) (20395 sequences) protein database for protein identification. At least 3 matched peptides per protein, representing 38.7% of the total sequence coverage, and a false discovery rate (FDR) of ≤ 5% were used as a cut-off. Exponentially modified Protein Abundance Index (emPAI) and % emPAI were calculated as described in **Supplementary Table 1**. Gene Ontology (GO) analysis was performed to classify proteins based on molecular functions, biological processes and cellular localization. String-DB was used for interactomics analysis (29).

### 2.7 Western blot validation of S100A8 abundance

In order to validate the presence of S100A8 in pSpA SF, were performed SDS-PAGE (15% gels) of SF pool (1/20 dilution, 20 μg total protein) and individual pSpA synovial fluids (1/20 dilution). The gel was electroblotted. Immunoreactivity with anti-human S100A8/S100A9 mouse IgG (Biolegend) was scanned using the Odyssey Infrared Imaging System (LI-COR, Lincoln, NE, U.S.A.). Western blot images were analyzed using a C-Digit scanner and software (Li-Cor, Lincoln, Nebraska, USA).

### 2.8 S100A8 expression in FLS

SW-982 FLS were incubated on coverslips, fixed in 4% paraformaldehyde and incubated with anti-human S100A8/S100A9 mouse IgG (Biolegend) (1:100 in 0.2% Triton X-100/saline), and anti‐mouse IgG‐Alexa‐Fluor‐594 (1:400, in Triton X-100/saline). The nuclei were stained with DAPI (0.3 nm in saline). The cells were examined using the microscope Axiovert X40 (Zeiss, Oberkochen, Germany).

### 2.9 Statistical analysis

Statistical significance was assessed by one-way analysis of variance (ANOVA) or two-way ANOVA followed by Tukey or Bonferroni multiple comparison test as appropriate. All experiments were performed at least twice, in some cases as 3 or 4 independent experiments. At least triplicates were included in each independent experiment. A p value < 0.05 was considered statistically significant. Data were analyzed using GraphPad Prism 5.0 software (GraphPad Software, La Jolla, CA, USA).

## 3 Results

### 3.1 Peripheral SpA synovial microenvironment induces early IL-6 production by FLS

**Figure 1:**
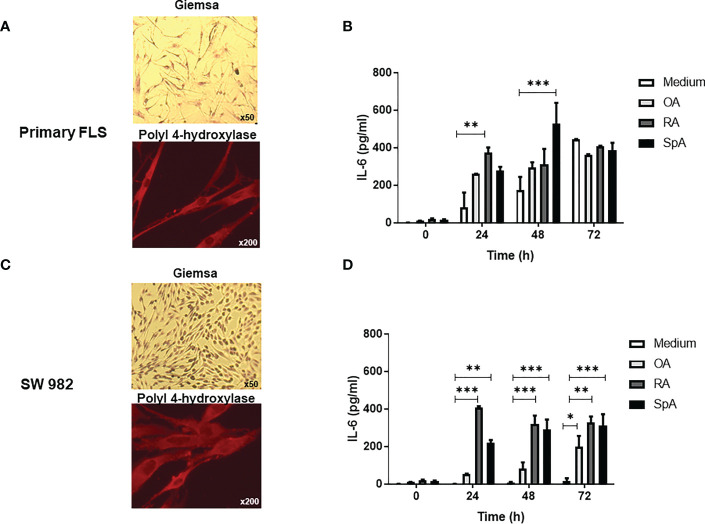
IL-6 secretion by primary and SW982 FLS stimulated with peripheral SpA synovial fluid. Morphology (Giemsa, original magnification x50) and prolyl-4-hydroxylase immunostaining (original magnification x200) of primary **(A)** or SW982 **(C)** FLS. IL-6 levels in the supernatants of primary **(B)** or SW982 **(D)** FLS incubated with medium, SF pool of OA, RA or peripheral SpA patients. Representative images **(A, C)** and data **(C, D)** from three independent experiments are shown. Values are presented as the mean ± SEM. *: p<0.05, **: p<0.01, ***: p<0.001 by the two-way ANOVA followed by Bonferroni multiple comparison test. OA, osteoarthritis; RA, rheumatoid arthritis; SpA, spondyloarthritis.

To investigate the effect of the synovial pSpA microenvironment on FLS, we first cultured primary or SW987 FLS, which presented fibroblast morphology and were positive for prolyl-4-hydroxylase (**Figures 1A, C**) and the purity of FLS was demonstrated by FACS (**Supplementary Figure 1**). Then, we incubated these FLS with OA, RA or SpA SF pools and measured the IL-6 production in the culture supernatant obtained at different times (**Figures 1B, D**). Although significant IL-6 levels are present in the SF of RA and SpA patients (**Supplementary Figure 2**), they are insignificant when diluted in the medium at the initial time of the cell cultures (**Figures 1B, D**). At 24 h or 48 h, RA- or SpA-stimulated-FLS secreted higher IL-6 compared with non-stimulated primary FLS (**Figure 1B**). A similar result was obtained in SW982 FLS (**Figure 1D**).

### 3.2 IL-6 controls the MMP-9 expression and activity induced by peripheral SpA synovial microenvironment in FLS *via* JNK/p38 MAPKs and JAK signaling

**Figure 2:**
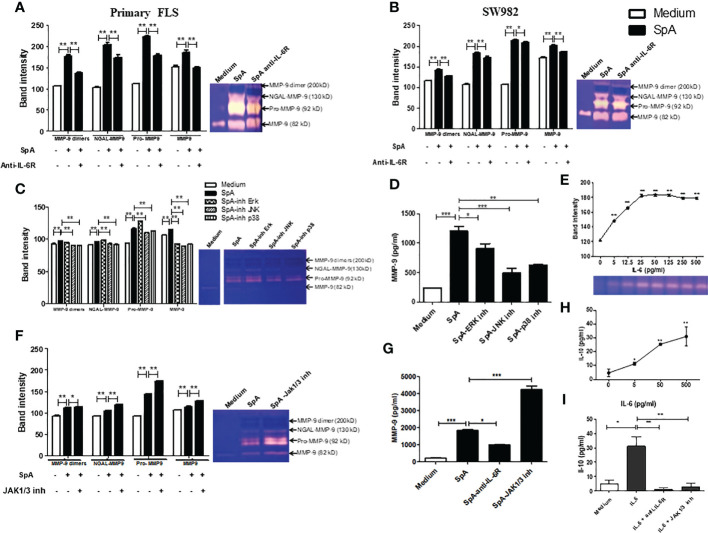
IL-6 controls the MMP-9 expression and activity induced by the peripheral SpA synovial microenvironment in FLS. Gelatin zymogram of 48h-supernatants of primary **(A)** or SW982 **(B)** FLS stimulated by SpA synovial fluids in the absence or the presence of anti-IL-6R (tocilizumab). Gelatin-zymography of supernatant of SW982 FLS pretreated with MAPKs **(C)** or JAK1/3 **(F)** inhibitors and their respective MMP-9 protein levels **(D, G)**. Gelatin-zymography of supernatant of IL-6-stimulated SW982 FLS **(E)**. IL-10 levels secreted by SW982 FLS stimulated with IL-6 **(H)** or IL-6 in the presence of anti-IL-6R or JAK1/3 inhibitor **(I)**. Data of at least two independent experiments. Values are presented as the mean ± SEM. *: p<0.05; **: p<0.01, ***: p<0.001.

We investigated whether pSpA-induced IL-6 could affect the MMP-9 activity of FLS. Thus, the gelatinase activity of MMP-9 forms was analyzed in the supernatants by gelatin-zymography. After pSpA SF stimulation of primary FLS, a significant increase in the activity of several bands (200 kD, MMP-9-dimer; 130 kD, NGAL-MMP-9 complex; 92 kD, Pro-MMP-9; and 82 kD, MMP-9 monomer) was detected. The presence of anti-IL-6R antibody (tocilizumab) significantly reduced the proteolytic activity of all MMP-9 forms (**Figure 2A**). Similar results were observed when we repeated the experiments for SW982 FLS (**Figure 2B**). Indeed, the ELISA analysis revealed that SpA SF increased the MMP-9 protein levels in the supernatants of SW982 FLS, which were significantly reduced in the presence of the anti-IL-6R antibody (**Figure 2G**). Likewise, we confirmed that IL-6 led to a dose-dependent induction of MMP-9 activity (**Figure 2E**). As we verified that both the primary FLS and SW982 cells respond in a similar way to synovial fluid stimulation, we decided to continue the study only with the SW982 cell line.

In the canonical IL-6/IL-6Rα induced-signaling, JAK activation triggers STAT and MAPKs signaling pathways (13). To prove that the synovial microenvironment of pSpA induces MMP-9 activity through MAPKs signaling, we pre-incubated SW982 FLS with various MAPKs inhibitors. We found that mainly JNK and p38 MAPKs inhibition significantly reduced MMP-9 activity (**Figure 2C**) and MMP-9 protein level (**Figure 2D**), suggesting that pSpA-induced MMP-9 expression in FLS is dependent on JNK and p38 MAPKs activation. However, when we pre-treated SW982 FLS with a JAK1/3 inhibitor (tofacitinib), pSpA SF unexpectedly increased MMP-9 activity (**Figure 2F**) and MMP-9 protein levels (**Figure 2G**). Therefore, we explore whether IL-6 modulates the induction of MMP-9 by JAK-dependent IL-10 expression. We demonstrated that IL-6 co-triggered MMP-9 (**Figure 2E**) and the anti-inflammatory cytokine IL-10 (**Figure 2H**). Moreover, IL-6-induced IL-10 secretion by SW982 FLS was mediated through JAK signaling (**Figure 2I**).

### 3.3 Pro-inflammatory cytokines and protein components of peripheral SpA synovial microenvironment promote IL-6 secretion by FLS

**Figure 3:**
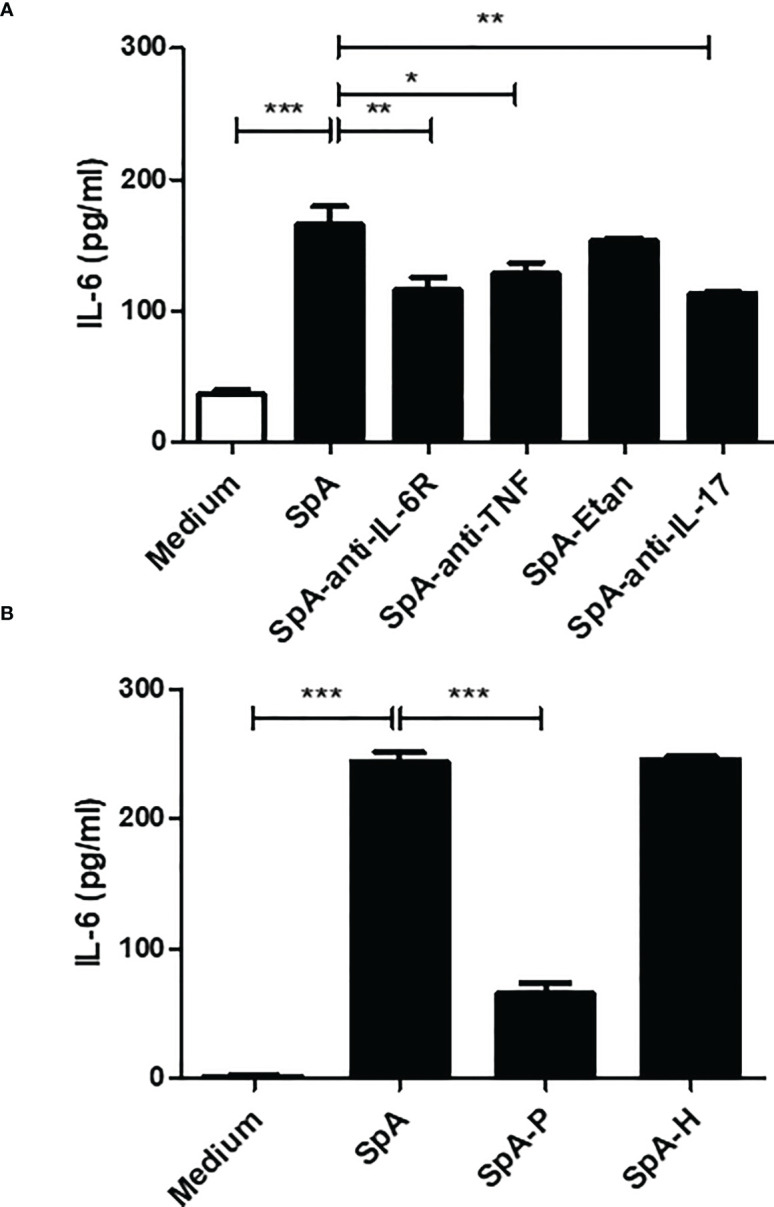
Synovial proteins promote IL-6 secretion by FLS. **(A)** SW982 FLS were incubated with a pool of peripheral SpA synovial fluid in the absence or presence of anti-IL-6R (tocilizumab), anti-TNF (infliximab), TNF inhibitor (etanercept, Etan) or anti-IL-17 (secukinumab). Non-stimulated FLS were used as a control. After 48h, IL-6 levels were measured in the culture supernatants. **(B)** SW982 FLS were stimulated with a pool of peripheral SpA SF pre-treated with proteinase K (SpA-P) or hyaluronidase (SpA-H). Data of at least two independent experiments are shown. Values are presented as the mean ± SEM. *: p<0.05; **: p<0.01, ***: p<0.001.

Pro-inflammatory cytokines, including tumor necrosis factor (TNF) and IL-17, induce IL-6 secretion by FLS of RA patients (30, 31). Moreover, TNF inhibition markedly decreases IL-6 levels in active SpA (32). In turn, IL-6 is a major cytokine-promoting Th17 differentiation (33). Thus, we examined whether synovial TNF, IL-17 and IL-6 itself are participating in the IL-6 production by FLS. We observed that the stimulation of SW982 FLS with SpA SF in the presence of anti-IL-6R (tocilizumab), anti-TNF (infliximab) or anti-IL-17 (secukinumab) significantly reduced IL-6 production by FLS (**Figure 3A**).

The SF comprises hyaluronic acid (HA) and proteins released from the synovial membrane and inflammatory cells (34). To investigate whether HA or proteins are involved in pSpA-induced IL-6 secretion, we stimulated SW982 FLS with SpA-P or SpA-H. In contrast to SpA-H, stimulation with SpA-P markedly reduced the IL-6 production by FLS (**Figure 3B**).

### 3.4 Synovial fluid proteins in peripheral SpA

**Figure 4:**
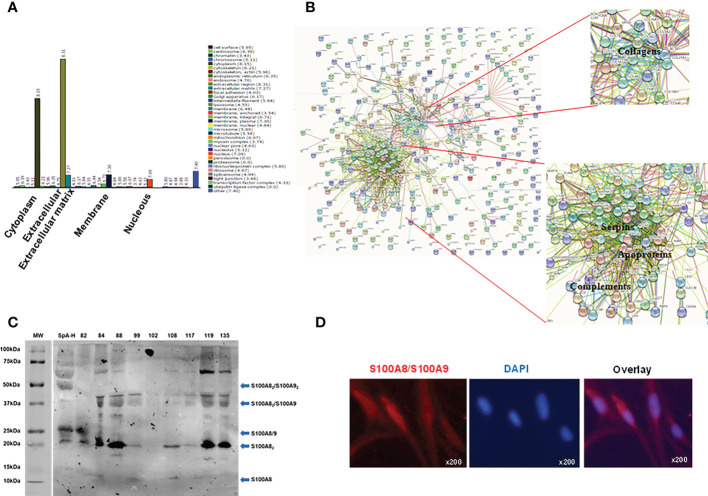
Proteins identified in peripheral SpA synovial fluid and S100A8 detection. **(A)** Gene ontology identified 355 proteins in a pool of 9 SF of peripheral SpA pre-treated with hyaluronidase (the majority were extracellular and cytoplasmatic proteins). **(B)** Interactomics analysis by String-DB shows the most abundant proteins: complement proteins, apoproteins, serpins and collagens. **(C)** Western blot analysis showing immunoreactivity with the anti-S100A8/S100A9 antibody. Molecular weight (MW) markers, the SpA-H pool (1/20 dilution), and individual SpA synovial fluids (1/20 dilution) were evaluated. Arrows indicate the observed S100A8 and S100A9 combinations. **(D)** Immunofluorescent detection of S100A8 (representative S100A8-positive fibroblasts), original magnification x200.

Proteomic studies have been useful for obtaining global protein profiling in human body fluids, including SF (35). Therefore, we performed a proteomic study of SpA-H which led to the identification of 355 proteins. The majority of proteins were cytoplasmatic and extracellular (**Figure 4A**). From the list of the total proteins identified, we focused on complement proteins, apoproteins, serpins, collagens (**Figure 4B**, **Supplementary Table 1**), and many novel proteins, including S100A8. This last one ranked 58 in the most abundant proteins (% emPAI 0.17) (**Supplementary Table 1**). After complement components and immunoglobulin chains, S100A8 was the first protein that, as an alarmin, plays a key role in the development of inflammation; moreover, it has been suggested that S100/S100A9 heterodimer may represent a good therapeutic target in psoriasis and psoriatic arthritis (36). Therefore, we focused on S100A8. First, the presence of S100A8 in SpA SF was validated by Western blot (**Figure 4C**). We detected a higher amount of S100A8 homodimer (20 kDa). Moreover, we found components migrating with a mass equivalent to S100A8/S100A9 heterodimer (25kDa), heterotrimer S100A8_2_S100A9 (35 kDa) and heterotetramer (47.5 kDa) (**Figure 4C**). Secondly, we observed intracellular S100A8 expression in SW982 FLS (**Figure 4D**).

### 3.5 S100A8 of the synovial peripheral SpA microenvironment is involved in inducing IL-6 and MMP-9 secretion by FLS

**Figure 5:**
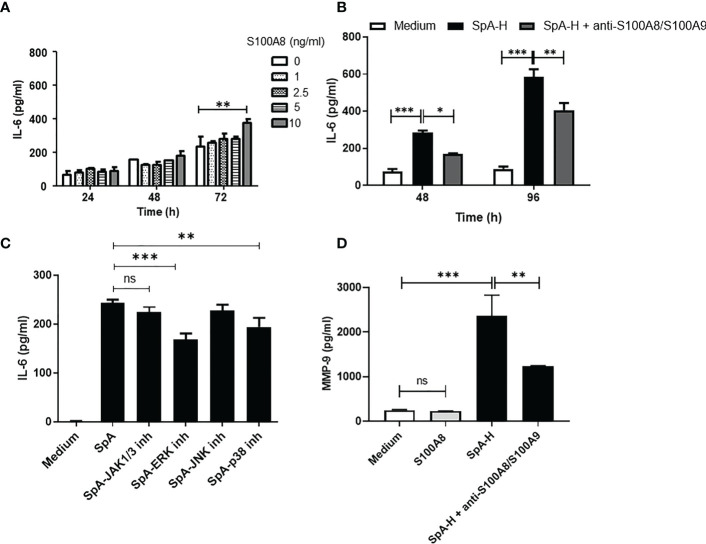
Synovial S100A8 induces IL-6 and MMP-9 secretion by FLS. **(A)** IL-6 levels secreted by SW982 FLS stimulated with S100A8 (1-10 ng/ml) or **(B)** with SpA SF pre-treated with hyaluronidase in the absence or presence of anti-S100A8/S100A9 antibody (5 μg/ml). In **(C)**, SW982 FLS were pre-treated with JAK1/3 or MAPKs inhibitors. **(D)** MMP-9 protein levels secreted by SW982 FLS stimulated with S100A8 (10 ng/ml) or SpA-H in the absence or presence of anti-S100A8/S100A9 antibody. Data of at least two independent experiments are shown. Values are presented as the mean ± SEM. *: p<0.05; **: p<0.01, ***: p<0.001, ns: non-significant.

As an alarmin, S100A8 is released passively by necrotic cells or by active secretion from activated immune cells. Extracellular S100A8 activates inflammation by inducing the secretion of inflammatory cytokines (37). Therefore, we explored whether S100A8 affects IL-6 secretion by FLS. Treatment of SW982 FLS with recombinant S100A8 resulted in a dose- and time-dependent increase in IL-6 levels (**Figure 5A**). To confirm this effect in SpA SF, we stimulated FLS in the presence of a selective anti-S100A8/S100A9 antibody, which significantly reduced IL-6 levels in 48h- or 96h- supernatants compared with control FLS (**Figure 5B**). In addition, S100A8, in similar concentration and time of stimulation, participated in the IL-23 production by FLS (**Supplementary Figure 3**) but not in TNF secretion (data not shown). ERK and p38 MAPKs signaling has been demonstrated to be activated by S100A8 binding (38). In contrast to tofacitinib, the ERK and p38 MAPKs inhibitors decreased pSpA-induced IL-6 secretion (**Figure 5C**). Finally, to associate this pro-inflammatory circuit with articular damage, we studied whether synovial S100A8 impacts MMP-9 secretion by FLS. S100A8 did not induce direct MMP-9 secretion. However, we found lower MMP-9 levels in the 48h-supernatants of FLS stimulated with SpA-H in the presence of anti-S100A8/S100A9 antibody (**Figure 5D**).

## 4 Discussion

In this study, we revealed a pro-inflammatory axis in the synovial microenvironment of peripheral SpA, which involves the S100A8 as a trigger of IL-6 and MMP-9 secretion by FLS. In addition, we identified a JAK-dependent mechanism controlling IL-6-induced MMP-9 production by FLS. To the best of our knowledge, this is the first study to describe molecular mechanisms that reveal the impact of the synovial milieu on FLS in peripheral SpA, which contributes to shedding light on the pathogenesis of this disease.

We had previously reported an increased IL-6 concentration in RA and SpA compared with OA patients (39). In the present work, we found that the RA or peripheral SpA synovial fluid provoke early secretion of IL-6 in primary and SW982 FLS. This suggests an evident positive pro-inflammatory course of the synovial milieu on FLS. Accordingly, IL-6 plays a role in the SpA pathogenesis, particularly in certain clinical phenotypes of aggressive and destructive peripheral arthritis, which has responded to IL-6 inhibition (40). In addition, MMPs cleave the cartilage components (18, 41), and IL-6 induces MMPs production in SpA (18). In fact, we showed that anti-IL-6 reduced MMP-9 activity and protein levels in the supernatants of SpA-stimulated FLS. Remarkably, we detected a reduction in the activity of all MMP-9 forms, including MMP-9-NGAL, which protects MMP-9 from degradation (17, 42). Notably, all cell types, including FLS, express gp130 (11), and SF contains high sIL-6R levels (14). Therefore, we argue that trans-activation mediates MMP-9 production by FLS. We found that SpA-induced MMP-9 secretion depends on JNK and p38 MAPKs activation. However, the inhibition of JAK1/3 enhanced MMP-9 activity and protein expression. These data support the necessity of clinical evaluation of tofacitinib in different SpA phenotypes (43). Since IL-6-induced IL-10 secretion was completely blocked by the JAK1/3 inhibitor, we suggest IL-10 as the IL-6-induced JAK-dependent suppressor of MMP-9. These findings are consistent with the anti-inflammatory role of IL-10 and a previous report on murine macrophages (13). Therefore, we speculate that feedback mechanisms may operate in the pSpA synovial milieu.

Even though the triggers and pathogenesis of pSpA are not yet completely understood, therapeutic agents targeting TNF or IL-17 inflammatory pathways have suppressed many clinical symptoms and signs of SpA, and they may be responsible for initiating and maintaining SpA inflammation (44, 45). In this work, we confirm a cytokine fine-tuning in the inflammation process of the SpA synovial microenvironment. Considering the complexity of SF, we wanted to explore additional mediators than cytokines that stimulate FLS for IL-6 production. We found that protein disruption potently attenuated IL-6 secretion. The protein compositions from pooled RA, SpA and OA SF have been compared (35, 46–48). In this work, we characterized the protein profile of the peripheral SpA SF pool used for FLS stimulation. We used two approaches to include in our analysis proteins in low abundance (48). The GO analysis classified most of the proteins as extracellular, in agreement with the fact that SF is an extracellular fluid. In addition, many proteins were in the cytoplasm group, and few in the membrane and nucleus groups, supporting a high degree of cellular infiltration in SF (47, 49). Overall, our analysis resulted in the identification of 355 proteins in the SpA SF proteome. The re-discovery of several proteins previously implicated in inflammatory arthritis and detected in PsA, such as S100A8, haptoglobin, Ig kappa chain and MMP-3, provided an internal validation of our analytical proteomic approach (48, 50). Among other proteins, we identified complement proteins, apoproteins, serpins, and collagens (**Supplementary Table 1**), which have already been described in the context of SpA (8, 47, 51). MMP-9 upregulation in SF of SpA patients has been reported (8). We detected single sequences originating from collagen type I (COL1A1, COL1A2) and type IV (COL4A2, COL4A4 and COL4A5), which are MMP-9 substrates. In agreement with PsA SF proteomics (48), we recognized MMP-3 in SpA SF, which has been identified as a biomarker for diagnosing SpA, and as a predictor of the response to TNF inhibitor therapy (41).

We did not detect, through proteomics analysis, the presence of S100A9. However, it could be present in a smaller proportion. In fact, when we analyzed the individual synovial fluid samples by WB using an anti-S100A8/S100A9 antibody for detection, we found components migrating with a mass equivalent to S100A8/S100A9 heterodimer (25kDa) (52, 53), and S100A8_2_S100A9 heterotrimer (35 kDa) (53). Besides, we observed the heterotetramer (47.5 kDa) that is formed in the presence of calcium and zinc (52, 54). Other types of interactions could explain the higher molecular weight bands. For example, it has been reported that S100A8/S1009 can bind to annexin VI, and a molecular weight of 72 kDa is observed (55). Nevertheless, the most intense band in the samples is around 20 kDa, which corresponds to the S100A8 homodimer, reflecting a higher proportion of this molecule association concerning others. In line, S100A8 was the most abundant protein in the synovial fluid of RA patients (56).

We detected intracellular S100A8/S100A9 expression in SW982 FLS. In line with this, S100A8/S100A9 was intensely expressed in the synovial sublining layer (21). Therefore, we may consider FLS as an S100A8 cellular source. Additionally, we found that S100A8 of SpA SF induces direct IL-6 (**Figure 5A**) and IL-23 (**Supplementary Figure 3**) production by FLS. Indeed, S100A8 has been considered an acute stage marker of inflammatory arthritis (48). We detected that ERK and p38 MAPKs pathways were involved in the SpA-induced IL-6 secretion by FLS. Similarly, S100A8 induced identical signal transduction pathways (38). On the other hand, S100A8 did not promote MMP-9 secretion (**Figure 5D**). However, neutralizing of the S100A8/S100A9 reduced significantly the levels MMP-9 on the supernatants of SpA-stimulated FLS (**Figure 5D**). Different reports suggest that the S100A8 homo-oligomeric as well as the hetero-oligomeric protein S100A8/S100A9 complexes, may play distinct roles in cell physiology (57–59). Therefore, a direct effect of S100A8/S100A9 on MMP-9 production by FLS cannot be excluded. In addition, it has been reported that S100A9 is a regulatory unit preventing S100A8 degradation and that S100A8 is the active component of the S100A8/S100A9 protein (56, 60). Since the S100A8/S100A9 heterodimer is a TLR4 ligand, the potential mechanism of FLS activation by the S100A8/S100A9 heterodimer is *via* TLR4-PI4K-NF-kB and MAPK signaling (21, 56). Based on our results, the alarmin S100A8 plays a pivotal role in amplifying the inflammation in the pSpA synovial milieu.

Taken together our results uncover a pro-inflammatory axis that associates S100A8 with IL-6 and MMP-9 production by FLS in the context of the real peripheral SpA synovial microenvironment. Moreover, S100A8 may be a suitable target for peripheral SpA treatment.

## Data availability statement

The original contributions presented in the study are included in the article/**Supplementary Material**. Further inquiries can be directed to the corresponding author. Proteomic data have been deposited in the public community-supported repository MassIVE (dataset: MSV000090846).

## Ethics statement

This study was approved by the Ethics Committee of the Institute of Biology and Experimental Medicine (IBYME, CONICET, Buenos Aires, Argentina) (CE002-2017). The patients provided their written informed consent to participate in this study.

## Author contributions

JA and SF performed experiments, analyzed the data and revised the manuscript. EC and MP carried out the proteomic experiments, analyzed and deposited the data in MassIVE. RB, AM, RP-H and HT collected the synovial fluids, analyzed the data and revised the manuscript. RE obtained the immunofluorescence images and revised the manuscript. MDG conceived and designed the research, carried out the experiments, analyzed the data, and wrote and revised the manuscript. MDG had full access to all of the data in the study and takes responsibility for the integrity of the data and the accuracy of the data analysis. All authors contributed to the article and approved the submitted version.
