# Supplementary figures and images for: S100A8 alarmin supports IL-6 and metalloproteinase-9 production by fibroblasts in the synovial microenvironment of peripheral spondyloarthritis
Source: Front Immunol. 2023 Jan 9;13:1077914. doi: 10.3389/fimmu.2022.1077914 (PMC9868917; doi:10.3389/fimmu.2022.1077914)

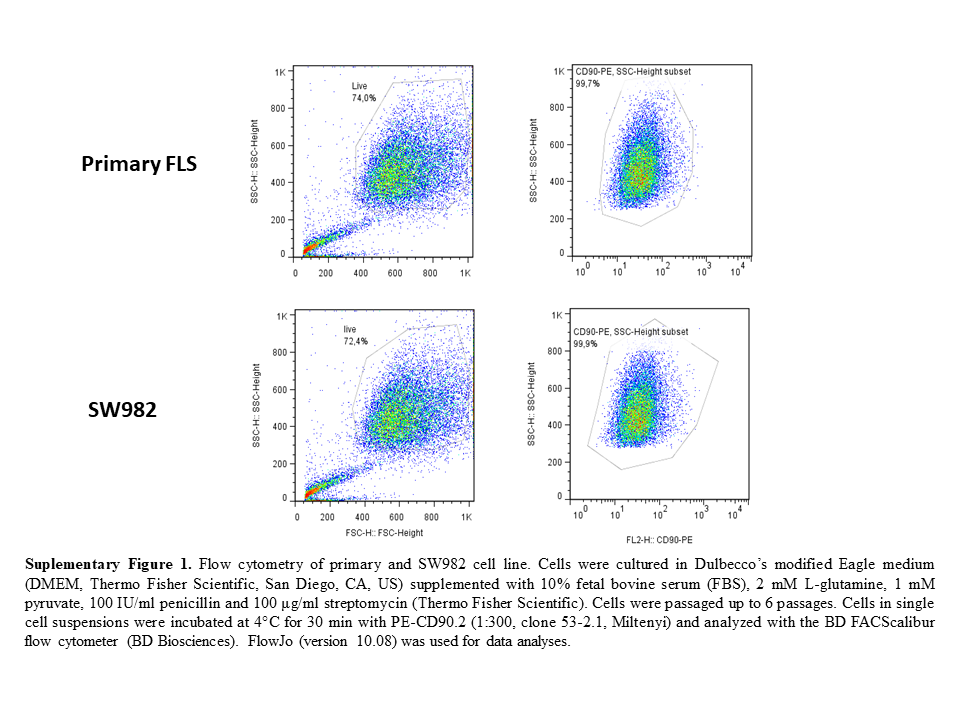

Supplement: Supplementary file 1 [file Image_1.tif]
